# Hyperfibrinolysis and fibrinolysis shutdown in patients with traumatic brain injury

**DOI:** 10.1038/s41598-022-23912-4

**Published:** 2022-11-09

**Authors:** Ryuta Nakae, Yasuo Murai, Takeshi Wada, Yu Fujiki, Takahiro Kanaya, Yasuhiro Takayama, Go Suzuki, Yasutaka Naoe, Hiroyuki Yokota, Shoji Yokobori

**Affiliations:** 1 Department of Emergency and Critical Care Medicine, Nippon Medical School Hospital, 1-1-5, Sendagi, Bunkyo-Ku, Tokyo, 113-8603 Japan; 2 Department of Neurological Surgery, Nippon Medical School Hospital, 1-1-5, Sendagi, Bunkyo-Ku, Tokyo, 113-8603 Japan; 3 Division of Acute and Critical Care Medicine, Department of Anesthesiology and Critical Care Medicine, Faculty of Medicine, Hokkaido University, N17W5, Kita-Ku, Sapporo-Shi, Hokkaido 060-8638 Japan; 4 Emergency and Critical Care Center, Kawaguchi Municipal Medical Center, 180, Nishiaraijuku, Kawaguchi-Shi, Saitama 333-0833 Japan; 5 Graduate School of Medical and Health Science, Nippon Sport Science University, 1221-1 Kamoshida-Cho, Aoba-Ku, Yokohama-Shi, Kanagawa 227-0033 Japan

**Keywords:** Brain injuries, Prognostic markers

## Abstract

Traumatic brain injury (TBI) is associated with coagulation/fibrinolysis disorders. We retrospectively evaluated 61 TBI cases transported to hospital within 1 h post-injury. Levels of thrombin-antithrombin III complex (TAT), D-dimer, and plasminogen activator inhibitor-1 (PAI-1) were measured on arrival and 3 h, 6 h, 12 h, 1 day, 3 days and 7 days after injury. Multivariate logistic regression analysis was performed to identify prognostic factors for coagulation and fibrinolysis. Plasma TAT levels peaked at admission and decreased until 1 day after injury. Plasma D-dimer levels increased, peaking up to 3 h after injury, and decreasing up to 3 days after injury. Plasma PAI-1 levels increased up to 3 h after injury, the upward trend continuing until 6 h after injury, followed by a decrease until 3 days after injury. TAT, D-dimer, and PAI-1 were elevated in the acute phase of TBI in cases with poor outcome. Multivariate logistic regression analysis showed that D-dimer elevation from admission to 3 h after injury and PAI-1 elevation from 6 h to 1 day after injury were significant negative prognostic indicators. Post-TBI hypercoagulation, fibrinolysis, and fibrinolysis shutdown were activated consecutively. Hyperfibrinolysis immediately after injury and subsequent fibrinolysis shutdown were associated with poor outcome.

## Introduction

The acute phase of traumatic brain injury (TBI) is known to be associated with disruptions of the coagulation/fibrinolysis system^1–3^. The haemorrhagic diathesis associated with hyperfibrinolysis has a significant impact on the mortality and functional outcome of TBI patients^2,4–6^. We previously investigated the time course of D-dimer, a marker of fibrinolysis, in patients with isolated TBI and an Abbreviated Injury Scale (AIS)^7^ of ≥ 3, and demonstrated that the plasma concentration of D-dimer was already abnormally high in 98.7% of patients within 1 h after injury, peaking at approximately 3 h after injury. Age and D-dimer levels were independent predictors of functional outcome at 3 months post-injury^8^. Fibrinolysis shutdown by plasminogen activator inhibitor-1 (PAI-1), which is synthesised by vascular endothelial cells, occurs after hyperfibrinolysis, but its pathogenesis is not well understood, and there have been no reports on its time course or relationship to long-term outcome. A deeper understanding of the pathogenesis of fibrinolysis shutdown is critically important, as it relates to the use of anti-fibrinolytic agents and thrombus formation. We hypothesised that hyperfibrinolysis and subsequent fibrinolysis shutdown may be associated with long-term prognosis.

Here, we evaluated the time course of coagulation abnormalities and subsequent fibrinolysis and fibrinolysis shutdown in the acute phase of TBI and their relationship to long-term outcome, focusing on blood biomarkers.

## Methods

### Ethical approval and consent to participate

All procedures performed in the study involving human participants were in accordance with the 1964 Helsinki declaration and its later amendments or comparable ethical standards. The study was approved by the Central Ethics Committee of Nippon Medical School (#M-2021-025, 16 February, 2022) and Ethics Committee of Kawaguchi Municipal Medical Center (#2021-35, 7 March, 2022), and the need for informed consent was waived due to the retrospective nature of the study.

### Patients

We studied the demographic, clinical, and radiological data of TBI patients admitted to Kawaguchi Municipal Medical Center from April 2018 to September 2021. Cases were included if the diagnosis was isolated TBI with intracranial AIS ≥ 3 and extracranial AIS < 3, as described previously^1,3,8^.

The diagnosis of TBI was established from cranial computed tomography (CT) findings. Intracranial and extracranial AIS and CT scans were independently evaluated by intensivists and neurointensivists (Y.F., G.S., Y.N.) at the study institution. Exclusion criteria were lack of information about the time of injury, first blood draw > 1 h after injury, age < 16 years, presence of diseases or drugs that affect coagulation and fibrinolysis parameters such as hepatic failure, anticoagulant medication, cardiopulmonary arrest prior to admission or on arrival, and withdrawal from active treatment.

Case data, including age, sex, Glasgow Coma Scale (GCS) score at admission, AIS^7^, administration of tranexamic acid (TXA), and amount of fresh frozen plasma (FFP) transfused within 7 days of injury were collected. CT scans at admission and afterwards were independently evaluated and the type of head injury was classified based on radiological findings as acute subdural hematoma (ASDH), acute epidural hematoma (AEDH), intracerebral hematoma and contusion (ICH), and traumatic subarachnoid haemorrhage (TSAH). The coagulation marker thrombin-antithrombin III complex (TAT), the fibrinolysis marker D-dimer, and the fibrinolysis inhibition marker PAI-1, as well as platelet count, prothrombin time (PT), activated partial thromboplastin time (APTT) were assessed over time. Blood samples were analysed upon arrival at the emergency department to determine the initial (within 1 h after injury) TAT, D-dimer, and PAI-1 levels, as well as platelet count, PT and APTT. The tests were repeated at 3 h, 6 h, 12 h, 1 day, 3 days, and 7 days after injury for a total of seven time points.

### Management of TBI

Immediately upon arrival at the emergency room, the patients were treated according to the Guidelines for the Management of Head Injury, 4th edition, of the Japan Society of Neurotraumatology^9^. All patients underwent brain CT after detailed neurological evaluation and initial stabilization. A second CT scan was performed within 3 h of admission and whenever there were signs of worsening clinical symptoms or increased intracranial pressure.

### Assessment of coagulation/fibrinolysis parameters

Blood samples were drawn into ethylenediaminetetraacetic acid (EDTA) plasma and citrate. D-dimer was measured by a latex immunoassay method (LIAS Auto D-dimer Neo®, Sysmex Corp., Kobe, Japan). TAT was measured by a chemiluminescent enzyme immunoassay method (STACIA CLEIA TAT®, LSI Medience Corp., Tokyo, Japan). PAI-1 was measured by a latex immunoassay method (LPIA-tPAI test®, LSI Medience Corp., Tokyo, Japan). Platelet count was measured by a DC sheath flow detection method (Cellpack II® and SE sheath II®, Sysmex Corp., Kobe, Japan). PT was measured by a coagulating time method (Dade Innovin®, Sysmex Corp., Kobe, Japan). APTT was measured by a coagulating time method (Thrombocheck APTT-SLA®, Sysmex Corp., Kobe, Japan).

### Statistical analysis

Data are presented as number (%) or median [interquartile range (IQR)]. The multiple imputation method was used for missing data^10,11^. Patients were divided into two groups according to the extended Glasgow Outcome Scale (GOS-E)^12,13^ at 6 months post-injury. The GOS-E is divided into the following eight categories: (1) dead, (2) vegetative state, (3) lower body severe disability, (4) upper body severe disability, (5) lower body moderate disability, (6) upper body moderate disability, (7) lower body good recovery, and (8) upper body good recovery. The good outcome group consisted of cases with a GOS-E of 6–8, and the poor outcome group consisted of patients with a GOS-E of 1–5. The GOS-E was assessed by neurointensivists (Y.F. and T.K.) at the study institution, who contacted patients, patient families, and hospitals to which patients were transferred from our hospital after discharge, by telephone and mail. Demographic, clinical, and radiological parameters for both groups were analysed using Student’s *t*-test, the Mann–Whitney *U*-test, or the *χ*^*2*^ test for continuous normal, continuous non-normal, and dichotomous data, respectively. A paired *t*-test was used to determine whether there were a statistically significant differences in plasma levels of TAT, D-dimer, and PAI-1 and platelet count, PT and APTT at each time point. A generalised linear mixed model (GLMM) was used to compare the distributions of plasma levels of TAT, D-dimer, and PAI-1 and platelet count, PT and APTT between the good outcome and the poor outcome groups at the seven measurement time points. Spearman’s rank correlation coefficient was used to investigate the correlation of plasma D-dimer and PAI-1 levels. Multivariate logistic regression analysis by the forced entry method was conducted to identify prognostic coagulation and fibrinolysis parameters at each time point^14^. A value of *p* < 0.05 was considered statistically significant. All statistical analyses were performed using commercial software (SPSS Version 25.0®; IBM Corp., Armonk NY, USA).

## Results

A total of 61 consecutive TBI cases were included in the study. There were 122 missing values (5.3%) in this study’s dataset, which were complemented by the multiple imputation method. Demographic, clinical, and radiological characteristics are summarised in Table 1. ASDH was found in 49 patients (80.3%), AEDH in 10 patients (16.4%), ICH in 52 patients (85.2%), and TSAH in 58 patients (95.1%) (some patients had more than one diagnosis). The good outcome group consisted of 30 cases (49.2%) and the poor outcome group consisted of 31 cases (50.8%). Seven patients died between 1 and 3 days after injury and five patients died between 3 and 7 days after injury due to TBI. Age was significantly lower in the good outcome group than in the poor outcome group [median 48 years (IQR 32–66 years) vs. 78 years (IQR 59–82 years), *p* < 0.001]. There was no difference in gender between the two groups. The good outcome group had higher GCS scores at admission [median 13 (IQR 11–15) vs. 6 (IQR 4–13), *p* < 0.001], lower AIS-head [median 4 (IQR 3–5) vs. 5 (IQR 5–5), *p* < 0.001], lower incidence of ASDH (63.3% vs. 96.8%, *p* = 0.009), and lower volume of FFP transfusion [median 0 mL (IQR 0–0 mL) vs. 0 mL (IQR 0–1200 mL), *p* = 0.008] than the poor outcome group. There was no difference in the rate of TXA use between the two groups.

**Table 1:** Initial demographic, clinical, and radiologic characteristics of the study population.

	Total (n = 61)	Good outcome group (n = 30)	Poor outcome group (n = 31)	*p*
**Demographic data**				
Age, median (IQR), year	65 (41–80)	48 (32–66)	78 (59–82)	** < 0.001**
Male, n (%)	38 (62.3)	19 (63.3)	19 (61.3)	0.87
**Clinical scores**				
GCS score, median (IQR)	12 (6–14)	13 (11–15)	6 (4–13)	** < 0.001**
AIS-head, median (IQR)	5 (4–5)	4 (3–5)	5 (5–5)	** < 0.001**
**CT findings**				
ASDH, n (%)	49 (80.3)	19 (63.3)	30 (96.8)	**0.009**
AEDH, n (%)	10 (16.4)	7 (23.3)	3 (9.7)	0.15
ICH, n (%)	52 (85.2)	23 (76.7)	29 (93.5)	0.07
TSAH, n (%)	58 (95.1)	28 (93.3)	30 (96.8)	0.54
**Treatment**				
TXA, n (%)	21 (34.4)	11 (36.7)	10 (32.3)	0.72
FFP, median (IQR), mL	0 (0–720)	0 (0–0)	0 (0–1200)	**0.008**

Good outcome group: extended Glasgow Outcome Scale (GOS-E) of 6–8; Poor outcome group: GOS-E of 1–5.

*IQR* interquartile range, *GCS* Glasgow Coma Scale, *AIS* Abbreviated Injury Scale, *ASDH* acute subdural hematoma; *AEDH* acute epidural hematoma, *ICH* intracerebral hematoma and contusion, *TSAH* traumatic subarachnoid hemorrhage, *TXA* tranexamic acid, *FFP* fresh frozen plasma.

All values are expressed as number (%) or median (first to third quartile).

Significant values are in bold.

### Time course of TAT, D-dimer, PAI-1, platelet count, PT, and APTT

**Figure 1:**
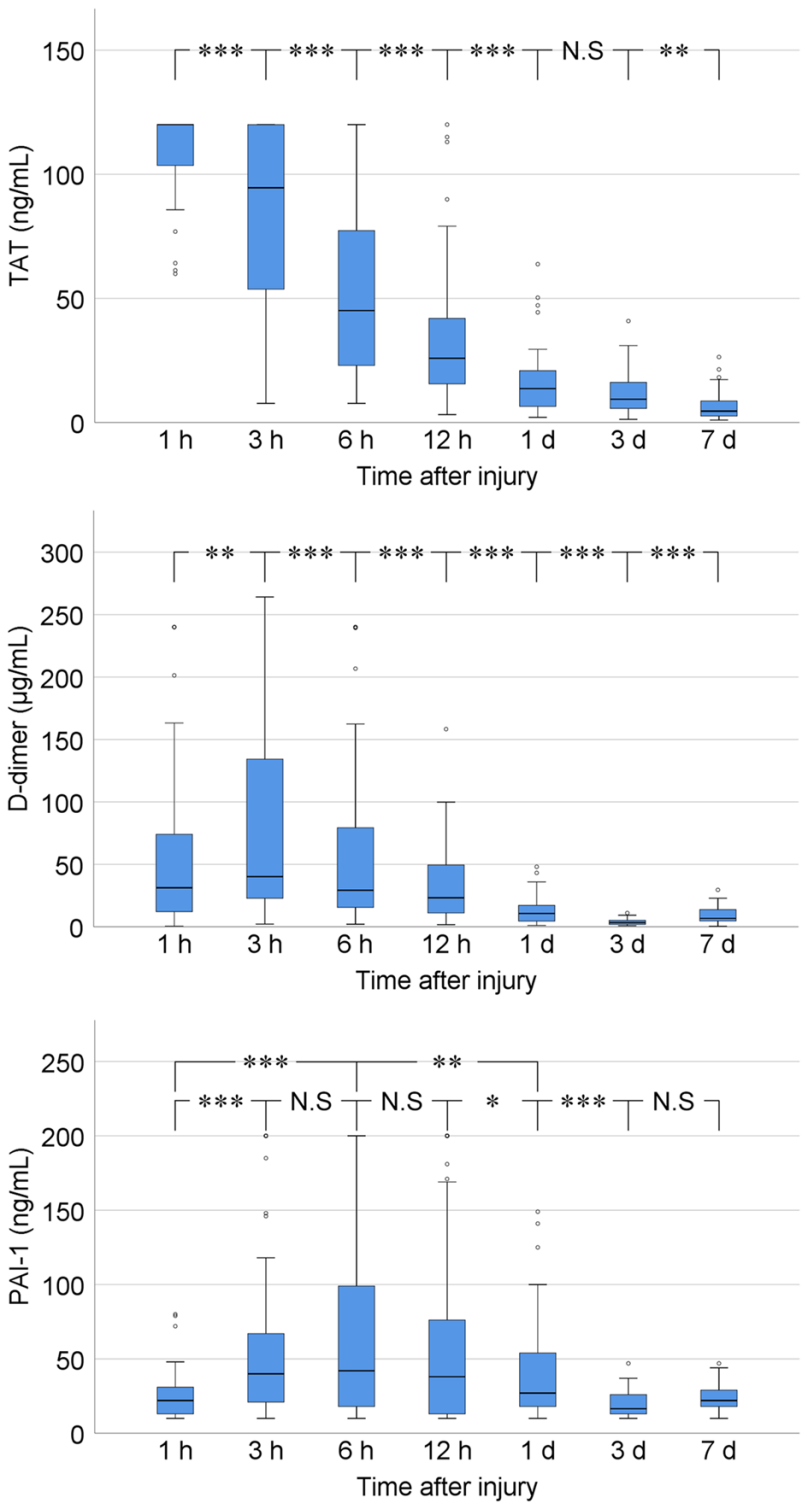
Boxplots showing plasma levels of thrombin-antithrombin III complex (TAT), D-dimer, and plasminogen activator inhibitor-1 (PAI-1) of all patients on admission and 3 h, 6 h, 12 h, 1 day, 3 days, and 7 days after traumatic brain injury. *p < 0.05, **p < 0.01, ***p < 0.001, N.S = Not Significant.

Figure 1 shows the time course of plasma TAT, D-dimer, and PAI-1 levels of all patients on admission and 3 h, 6 h, 12 h, 1 day, 3 days, and 7 days after TBI. Plasma TAT levels (normal range: 0.0–3.0 ng/mL) at admission were abnormally high in all patients. The median plasma level of TAT decreased rapidly and significantly from admission to 1 day after injury [1–3 h: *t* (60) = 4.531, *p* < 0.001; 3–6 h: *t* (60) = 7.753, *p* < 0.001; 6–12 h: *t* (60) = 4.907, *p* < 0.001; 12 h–1 day: *t* (60) = 5.665, *p* < 0.001], subsequently decreased insignificantly from 1 to 3 days after injury [*t* (53)  = 1.374, *p* = 0.17], and decreased significantly from 3 to 7 days after injury [t (48) = 2.657, *p* = 0.008].

The median plasma D-dimer level (normal range: 0.0–1.0 μg/mL) at admission was abnormally high in 60 (98.4%) of the 61 patients. It increased significantly from admission to 3 h after injury [*t* (60) = − 3.198, *p* = 0.01]. Three hours after injury, it decreased significantly up to 3 day after injury [3–6 h: *t* (60) = 4.709, *p* < 0.001; 6–12 h: *t* (60) = 3.629, *p* < 0.001; 12 h–1 day: *t* (60) = 4.880, *p* < 0.001; 1–3 days: *t* (53) = 4.798, *p* < 0.001], and subsequently increased significantly again from 3 to 7 days after injury [*t* (48) = − 6.444, *p* < 0.001].

The median plasma PAI-1 level (normal range: 0.0–50.0 ng/mL) at admission was within the normal range in 53 (86.9%) of the 61 patients. It increased significantly from admission to 3 h after injury [*t* (60) = − 5.302, *p* < 0.001]. The upward trend continued up to 6 h after injury [3–6 h: *t* (60) = − 1.550, *p* = 0.12; 1–6 h: *t* (60) = − 4.560, *p* < 0.001]. After 6 h post-injury, it decreased up to 3 days [6–12 h: *t* (60) = 0.640, *p* = 0.52; 12 h–1 day: *t* (60) = 2.309, *p* = 0.02; 6 h–1 day: *t* (60) = 3.193, *p* = 0.001; 1–3 days: *t* (53) = 3.868, *p* < 0.001], subsequently increasing insignificantly again from 3 to 7 days after injury [*t* (48) = − 0.400, *p* = 0.69].

Supplementary Fig. 1 shows the time course of platelet count, PT, and APTT of all patients on admission and 3 h, 6 h, 12 h, 1 day, 3 days, and 7 days after TBI. The median platelet count (normal range: 120–400 × 10^9^/L) decreased significantly from admission to 1 day after injury [1–3 h: *t* (60) = 5.881, *p* < 0.001; 3–6 h: *t* (60) = 3.157, *p* = 0.002; 6–12 h: *t* (60) = 2.048, *p* = 0.04; 12 h–1 day: *t* (60) = 2.486, *p* = 0.01], subsequently increased insignificantly from 1 to 3 days after injury [*t* (53) = − 0.224, *p* = 0.82], and increased significantly from 3 to 7 days after injury [*t* (48) = − 9.496, *p* < 0.001].

The median PT [normal range: 0.8–1.2 international normalised ratio (INR)] increased significantly from admission to 3 h after injury [*t* (60) = − 3.210, *p* = 0.003], subsequently decreased insignificantly from 3 h to 1 day after injury [3–6 h: *t* (60) = 1.426, *p* = 0.16; 6–12 h: *t* (60) = 1.639, *p* = 0.12; 12 h–1 day: *t* (60) = 1.612, *p* = 0.12], decreased significantly from 1 to 3 days after injury [*t* (53) = 5.582, *p* < 0.001], and increased significantly again from 3 to 7 days after injury [*t* (48) = − 4.436, *p* < 0.001].

The median APTT (normal range: 24–36 s) increased insignificantly from admission to 1 day after injury [1–3 h: *t* (60) = − 0.911, *p* = 0.36; 3–6 h: *t* (60) = − 0.138, *p* = 0.89; 6–12 h: *t* (60) = − 1.165, *p* = 0.24; 12 h–1 day: *t* (60) = − 0.766, *p* = 0.44], subsequently decreased significantly from 1 to 3 days after injury [*t* (53) = 4.270, *p* < 0.001], and decreased insignificantly from 3 to 7 days after injury [*t* (48) = − 0.595, *p* = 0.55].

### TAT, D-dimer, PAI-1, platelet count, PT, APTT and long-term outcome

**Figure 2:**
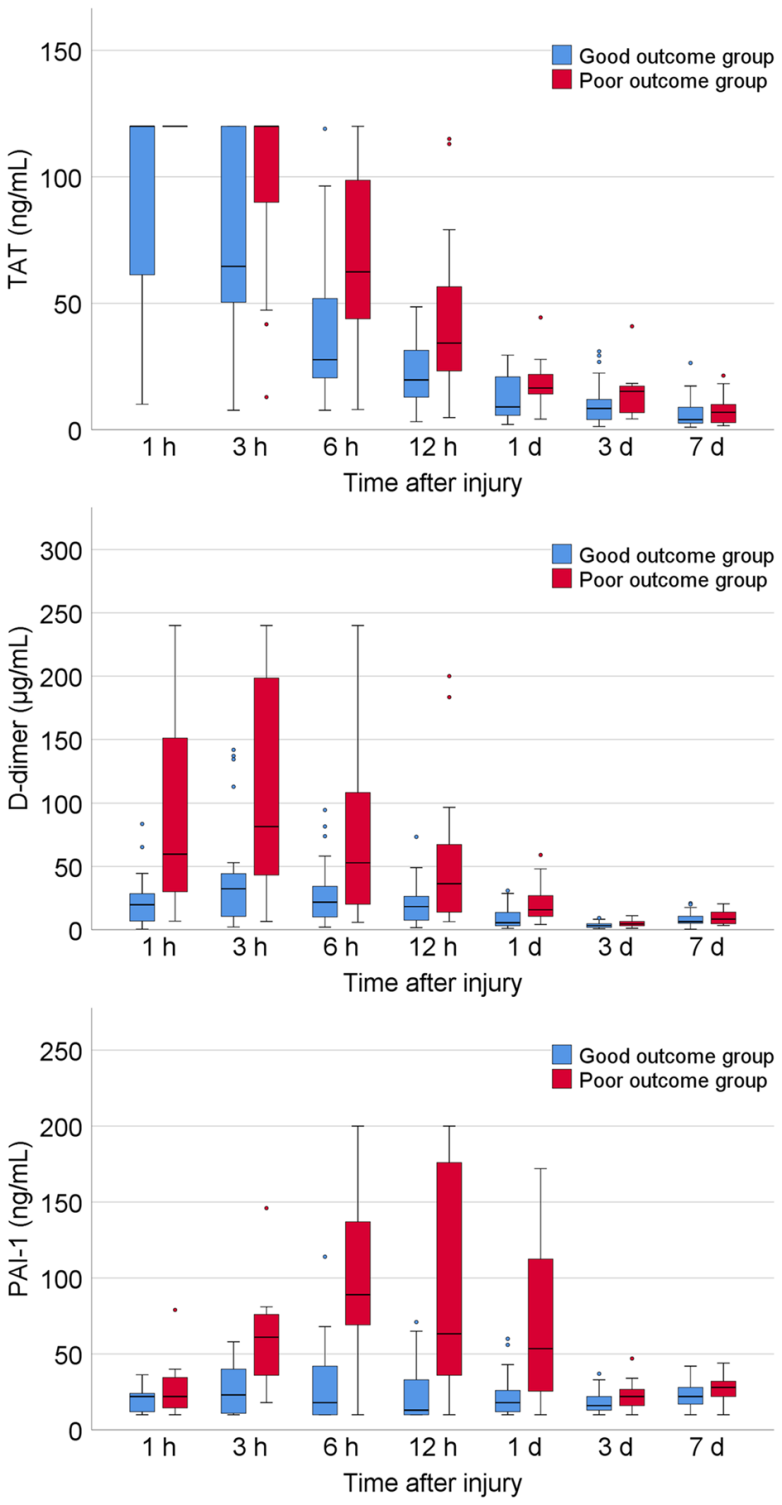
Boxplots showing plasma levels of thrombin-antithrombin III complex (TAT), D-dimer, and plasminogen activator inhibitor-1 (PAI-1) of cases with good outcome and poor outcome on admission and 3 h, 6 h, 12 h, 1 day, 3 days, and 7 days after traumatic brain injury.

The plasma levels of TAT, D-dimer, and PAI-1 were higher in the poor outcome group than in the good outcome group from the time of admission to 7 days after injury as analysed by GLMM (all *p* < 0.001) (Fig. 2). PT and APTT were higher in the poor outcome group than in the good outcome group (both *p* < 0.001), but platelet counts did not significantly differ between groups (*p* = 0.06) from the time of admission to 7 days after injury, as analysed by GLMM (Supplementary Fig. 2).

### Correlation between plasma D-dimer levels 3 h after injury and plasma PAI-1 levels 6 h after injury

**Figure 3:**
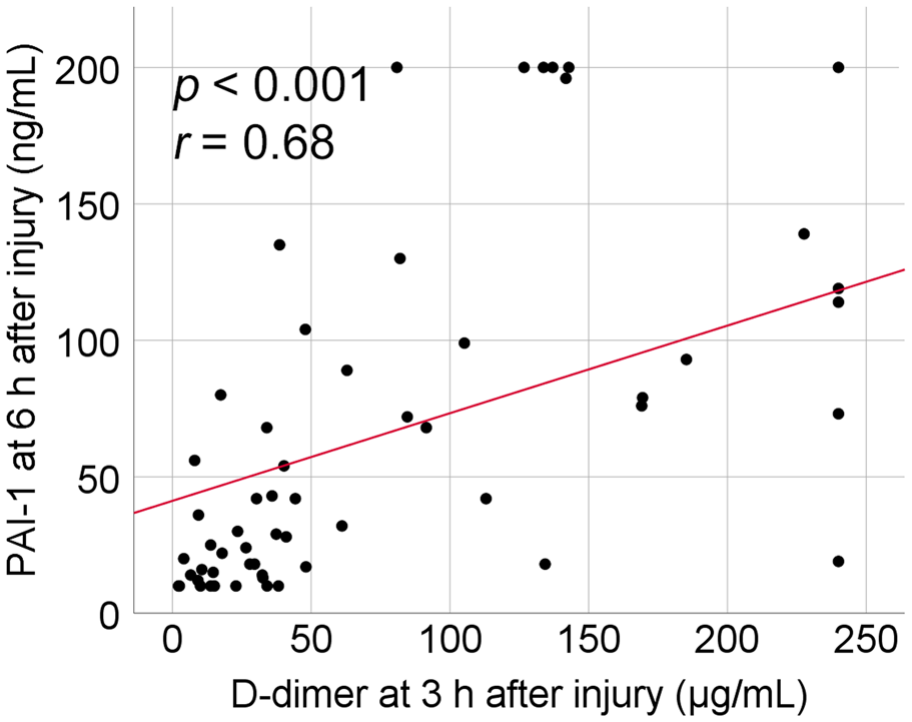
Correlation between plasma D-dimer level 3 h after injury and plasma PAI-1 level 6 h after injury.

Figure 1 shows that plasma D-dimer level as a biomarker of hyperfibrinolysis peaked 3 h after injury and plasma PAI-1 level as a biomarker of fibrinolysis shutdown peaked 6 h after injury. To investigate the correlation between hyperfibrinolysis and subsequent fibrinolysis shutdown, Spearman’s rank correlation coefficient between plasma D-dimer levels 3 h after injury and plasma PAI-1 levels 6 h after injury were calculated. Positive correlations were found between plasma D-dimer levels 3 h after injury and plasma PAI-1 levels 6 h after injury (*p* < 0.001, *r* = 0.68) (Fig. 3).

### Coagulation and fibrinolysis parameters as independent risk factors for poor prognosis

To evaluate independent risk factors at admission related to poor prognosis, multivariate logistic regression analysis was performed (Table 2). The explanatory variables were age^3,4,15–17^, GCS score^3,16,18–20^, AIS-head^3,4,18^, presence of ASDH^3,16,18,20^ and ICH^3,16,20,21^, and plasma levels of TAT, D-dimer, and PAI-1. The response variable was a good outcome with GOS-E of 6–8 or poor outcome with GOS-E of 1–5 at 6 months after injury. The results showed that independent risk factors at admission for poor prognosis were older age, presence of ASDH, and elevated D-dimer.

**Table 2:** Multivariate logistic regression analysis of initial variables as independent risk factors for poor prognosis.

Factor	Odds ratio (95% CI)	*p*
Age (10 years increments)	1.89 (1.16–3.08)	**0.01**
GCS score (1-point decrements)	1.19 (0.92–1.55)	0.18
AIS-head (1-point increments)	1.05 (0.17–6.72)	0.96
ASDH	22.35 (1.43–349.77)	**0.03**
ICH	6.83 (0.18–260.16)	0.30
TAT (10 ng/mL increments)	0.77 (0.53–1.10)	0.15
D-dimer (10 μg/mL increments)	1.92 (1.01–3.65)	**0.046**
PAI-1 (10 ng/mL increments)	1.05 (0.76–1.45)	0.75

*R*^*2*^ = 0.74.

*95% CI* 95% confidence interval, *GCS* Glasgow Coma Scale, *AIS* Abbreviated Injury Scale, *ASDH* acute subdural hematoma, *ICH* intracerebral hematoma and contusion, *TAT* thrombin-antithrombin III complex, *PAI-1* plasminogen activator inhibitor-1.

Significant values are in bold.

Multivariate logistic regression analysis was also performed using plasma levels of TAT, D-dimer, and PAI-1 from admission to 7 days after injury to identify reliable prognostic coagulation and fibrinolysis parameters at each time point after TBI (Table 3). Elevated D-dimer levels from admission to 3 h after injury and elevated PAI-1 levels from 6 h to 1 day after injury were significant negative prognostic indicators.

**Table 3:** Multivariate logistic regression analysis of coagulation/fibrinolysis parameters as independent risk factors for poor prognosis.

Factor	Admission		3 h after injury		6 h after injury		12 h after injury	
	Odds ratio (95% CI)	*p*	Odds ratio (95% CI)	*p*	Odds ratio (95% CI)	*p*	Odds ratio (95% CI)	*p*
TAT (10 ng/mL increments)	0.93 (0.74–1.17)	0.52	1.01 (0.82–1.26)	0.91	1.00 (0.77–1.31)	0.99	0.96 (0.71–1.30)	0.79
D-dimer (10 μg/mL increments)	1.65 (1.38–1.98)	**0.005**	1.16 (1.02–1.33)	**0.03**	1.13 (0.95–1.34)	0.18	1.28 (0.94–1.73)	0.12
PAI-1 (10 ng/mL increments)	1.03 (0.81–1.30)	0.82	1.17 (0.99–1.40)	0.07	1.20 (1.04–1.39)	**0.01**	1.31 (1.07–1.59)	**0.008**

Factor	1 day after injury		3 days after injury		7 days after injury			
	Odds ratio (95% CI)	*p*	Odds ratio (95% CI)	*p*	Odds ratio (95% CI)	*p*		
TAT (10 ng/mL increments)	1.11 (0.46–2.66)	0.82	0.81 (0.48–1.37)	0.42	1.49 (0.66–3.12)	0.36		
D-dimer (10 μg/mL increments)	1.47 (0.65–3.32)	0.36	15.45 (0.98–243.12)	0.05	1.28 (0.49–3.29)	0.62		
PAI-1 (10 ng/mL increments)	1.24 (1.00–1.53)	**0.047**	0.96 (0.75–1.23)	0.76	1.82 (0.94–3.55)	0.08		

Admission: *R*^*2*^ = 0.51; 3 h after injury: *R*^*2*^ = 0.48; 6 h after injury: *R*^*2*^ = 0.45; 12 h after injury: *R*^*2*^ = 0.51; 1 day after injury: *R*^*2*^ = 0.33; 3 days after injury: *R*^*2*^ = 0.25; 7 days after injury: *R*^*2*^ = 0.16.

*95% CI* 95% confidence interval, *TAT* thrombin-antithrombin III complex, *PAI-1* plasminogen activator inhibitor-1.

Significant values are in bold.

## Discussion

This study found that hypercoagulability, fibrinolysis, and fibrinolysis shutdown were activated one after another following TBI, with hypercoagulability peaking immediately after injury, fibrinolysis peaking 3 h after injury, and fibrinolysis shutdown peaking 6 h after injury. All of these coagulation and fibrinolytic changes were demonstrated to be activated at the time of TBI in patients with poor long-term outcomes, with plasma D-dimer levels from admission to 3 h after injury and plasma PAI-1 levels from 6 h to 1 day after injury being independent biomarkers of long-term outcome.

The cerebral arteries and astrocytes contain high concentrations of tissue factor (TF)^22,23^. After TBI, TF from injured brain tissue enters the systemic circulation, forms a complex with factor VIIa (FVIIa), activates the extrinsic pathway of blood coagulation, and causes excessive production of thrombin^24,25^. Thrombin converts fibrinogen to fibrin and forms thrombus, but some thrombin is inactivated on endothelial cells by forming a one-to-one complex with antithrombin III, a physiological inhibitor of thrombin. This complex is called TAT, and it is used as a marker of thrombin production in vivo to diagnose hypercoagulation. In this study, plasma TAT levels were abnormally high, peaking at admission in all TBI patients and decreasing over time, indicating that hypercoagulation after TBI is highest immediately after injury. In vivo studies have also reported the formation of microthrombi around brain contusions as early as 1 h after injury^26,27^. In addition, plasma TAT levels were higher in the poor outcome group than in the good outcome group, indicating that hypercoagulation is related to pathological progression in the acute phase of TBI^28^.

Hypercoagulation is followed by activation of fibrinolysis as a biological response. The fibrin clots formed by thrombin involvement are degraded by plasmin produced after plasminogen activation by tissue-type plasminogen activator (t-PA) or urokinase-type plasminogen activator (u-PA) to produce the fibrin degradation product, D-dimer^29,30^. In addition, tissue hypoperfusion associated with trauma results in the release of large amounts of t-PA from injured brain tissue, further activating fibrinolysis^31^. Thus, hyperfibrinolysis secondary to hypercoagulation and hyperfibrinolysis associated with direct t-PA release from the injured brain are the hallmarks of TBI and contribute to the bleeding diathesis. In the present study, plasma D-dimer levels were abnormally high in 98.4% of TBI patients at admission and peaked 3 h after injury. This suggests that hypercoagulation is followed by hyperfibrinolysis immediately after injury, peaking 3 h after injury. In addition, plasma D-dimer levels were higher in the poor outcome group than in the good outcome group and plasma D-dimer level from admission to 3 h after injury was an independent biomarker for long-term outcome. It has been reported that hyperfibrinolysis in the acute phase of TBI contributes to poor outcome through hematoma expansion^4,8,32,33^, which is consistent with the results of this study.

PAI-1 is a major endogenous inhibitor of fibrinolysis. Plasminogen activators, t-PA and u-PA, convert plasminogen into plasmin, which has proteolytic activity. Plasmin is an important enzyme in the fibrinolytic system, degrading fibrin clot into fibrin degradation products. PAI-1 is produced and secreted by vascular endothelial cells in response to hyperfibrinolysis, direct endothelial cell damage and inflammation associated with trauma, or tissue hypoxia due to thrombin production^34,35^. It inhibits fibrin clot lysis by inhibiting t-PA, u-PA, thrombomodulin, and activated protein C, thus reflecting fibrinolysis shutdown^35,36^. The timing of fibrinolysis shutdown after TBI has not been well-delineated, but in the present study, PAI-1 reached its peak 6 h after injury and correlated with the plasma D-dimer levels 3 h after injury, suggesting that the coagulation-fibrinolysis kinetics shifted to fibrinolysis shutdown after 3 h post-injury. Figure 2 shows that the plasma PAI-1 levels of most of the cases in the good outcome group remained within the normal range, while many of those in the poor outcome group had high levels from 3 h to 1 day after the injury. In addition, plasma PAI-1 levels from 6 h to 1 day after injury were an independent biomarker for long-term outcome. Gando et al.^34^ and Wada et al.^37^ showed that disseminated intravascular coagulation (DIC) with the fibrinolytic phenotype in the acute phase of TBI develops into DIC with the thrombotic phenotype (fibrinolysis shutdown) at a high rate and has a poor prognosis due to organ dysfunction from thrombus deposition. In a secondary analysis of previously collected data from a prospective cohort study, Rossetto et al.^38^ showed that the development of hypofibrinolytic state (fibrinolysis shutdown) after 24 h with rotational thromboelastometry (ROTEM®; TEM International GmbH, Munich, Germany) was associated with severe TBI patients and increased incidence of multiple organ failure and late mortality. The results of these studies support the findings of our study. Other reasons could be that PAI-1 itself also exerts neurotoxic, oxidative, and inflammatory effects. In a mouse model of TBI, it has been reported that administration of a PAI-1 antagonist reduced thrombus formation in the microvasculature around the cerebral cortex after trauma, inhibited neuronal apoptosis, and improved neurological function^39^. In a rat model of TBI, administration of a PAI-1 antagonist has been shown to have antioxidant and anti-inflammatory effects and to improve behavioural test outcomes^40^.

The viscoelastic devices (VEDs) such as thromboelastography (TEG®; Hemonetics, Braintree, MA, USA) and ROTEM® are an alternative to standard laboratory tests and are becoming more common. In severe trauma patients, evidence is increasing to show that the use of TEG® and ROTEM® reduces the need for blood transfusions and improves outcomes^41,42^. While the administration of VED is widely accepted for trauma patients, VED has been reported to have poor diagnostic sensitivity in hyperfibrinolytic states^43^, and its advantages for TBI patients are uncertain. A recent systematic review suggested that VED is useful as a rapid and comprehensive point-of-care alternative for identifying coagulopathy with significant consequences in TBI patients with intracranial haemorrhage^44^. In this study, we could not evaluate coagulation and fibrinolytic function using VED. Further studies are needed to confirm the efficacy of VED in TBI patients with hypercoagulability, fibrinolysis, and fibrinolysis shutdown sequentially.

The only evidence-based treatment for TBI with coagulation and fibrinolysis disruption is TXA, an antifibrinolytic agent, in a subset of patients with mild-to-moderate TBI^45^. The Clinical Randomisation of Antifibrinolytic in Significant Hemorrhage (CRASH-2) trial^46^, a large, international, multicentre, randomised, placebo-controlled trial investigating the effect of TXA on mortality and the need for transfusion in adult trauma patients with significant bleeding, showed that all-cause mortality was significantly lower in the TXA group than in the placebo group. A systematic review of two randomised controlled trials^47^, including the CRASH-2 trial, showed that TXA administration reduced mortality in TBI patients compared with placebo. However, the results appear to be highly dependent on the timing of TXA administration. It was reported that TXA administration within 3 h after injury was effective, but after 3 h, it appeared to increase mortality^48^. Based on these results, the protocol for administering TXA in the CRASH-3 trial, another large, international, multicentre, randomised, placebo-controlled trial examining the effect of TXA on head trauma-related mortality in TBI patients, was changed to within 3 h of trauma^45^. There is no consensus on the mechanism by which TXA administration after 3 h of injury adversely affects outcome. In this study, it was shown that the peak of hyperfibrinolysis after TBI was at 3 h after injury, with a subsequent shift to fibrinolysis shutdown, reaching its peak at 6 h after injury. Since PAI-1 is a major inhibitor of the fibrinolysis system, elevated PAI-1 levels may produce a hypofibrinolytic or prothrombotic state and contribute to microthrombus formation^35^. The time of the transition to a prothrombotic state has been unclear^48^, but it may be occur earlier than previously thought, and TXA administration more than 3 h after injury may have a negative prognostic effect.

## Limitations

Several limitations of this study warrant mention. First, the sample size was relatively small, which did not allow us to obtain sufficient power for statistical analysis. Second, there were many cases in which plasma TAT levels at admission was 120 ng/mL, the upper limit of measurement, which may have affected the statistical analysis. Third, the data were not designed to evaluate the impact of TXA and FFP administration and surgery, and which might have affected TAT, D-dimer, and PAI-1 after 3 h post-injury. Fourth, we did not investigate antifibrinolytic factors such as thrombin activatable fibrinolysis inhibitor or α2-antiplasmin in the present study. Fifth, coagulation and fibrinolytic function has not been evaluated by VEDs such as TEG and ROTEM. Finally, in Japan, the population is aging and the mean age of TBI patients is high^49^. As a result, it may appear that there is an overrepresentation of older patients with severe TBI.

## Conclusions

After TBI, hypercoagulation peaked immediately after injury, fibrinolysis peaked 3 h after injury, and fibrinolysis shutdown peaked 6 h after injury. All of these coagulation and fibrinolytic pathways were activated in patients with poor long-term outcomes. Plasma D-dimer levels from admission to 3 h after injury and plasma PAI-1 levels from 6 h to 1 day after injury were independent biomarkers for long-term outcome.

## Supplementary Information

Supplementary Figures.

## Data Availability

The datasets supporting the conclusions of this article will be provided based on reasonable request to the corresponding author.

## Abbreviations

AEDH: Acute epidural hematoma
AIS: Abbreviated Injury Scale
APTT: Activated partial thromboplastin time
ASDH: Acute subdural hematoma
DIC: Disseminated intravascular coagulation
FFP: Fresh frozen plasma
GCS: Glasgow Coma Scale
GLMM: Generalised linear mixed model
GOS-E: Extended Glasgow outcome scale
ICH: Intracerebral hematoma and contusion
INR: International normalised ratio
IQR: Interquartile range
PAI-1: Plasminogen activator inhibitor-1
PT: Prothrombin time
TAT: Thrombin-antithrombin III complex
TBI: Traumatic brain injury
TSAH: Traumatic subarachnoid haemorrhage
TXA: Tranexamic acid
VED: Viscoelastic device
