# Supplementary material for: Hyperfibrinolysis and fibrinolysis shutdown in patients with traumatic brain injury
Source: Sci Rep. 2022 Nov 9;12:19107. doi: 10.1038/s41598-022-23912-4 (PMC9646769; doi:10.1038/s41598-022-23912-4)
Supplement: Supplementary file 1 — Supplementary Figures. [file 41598_2022_23912_MOESM1_ESM.docx]

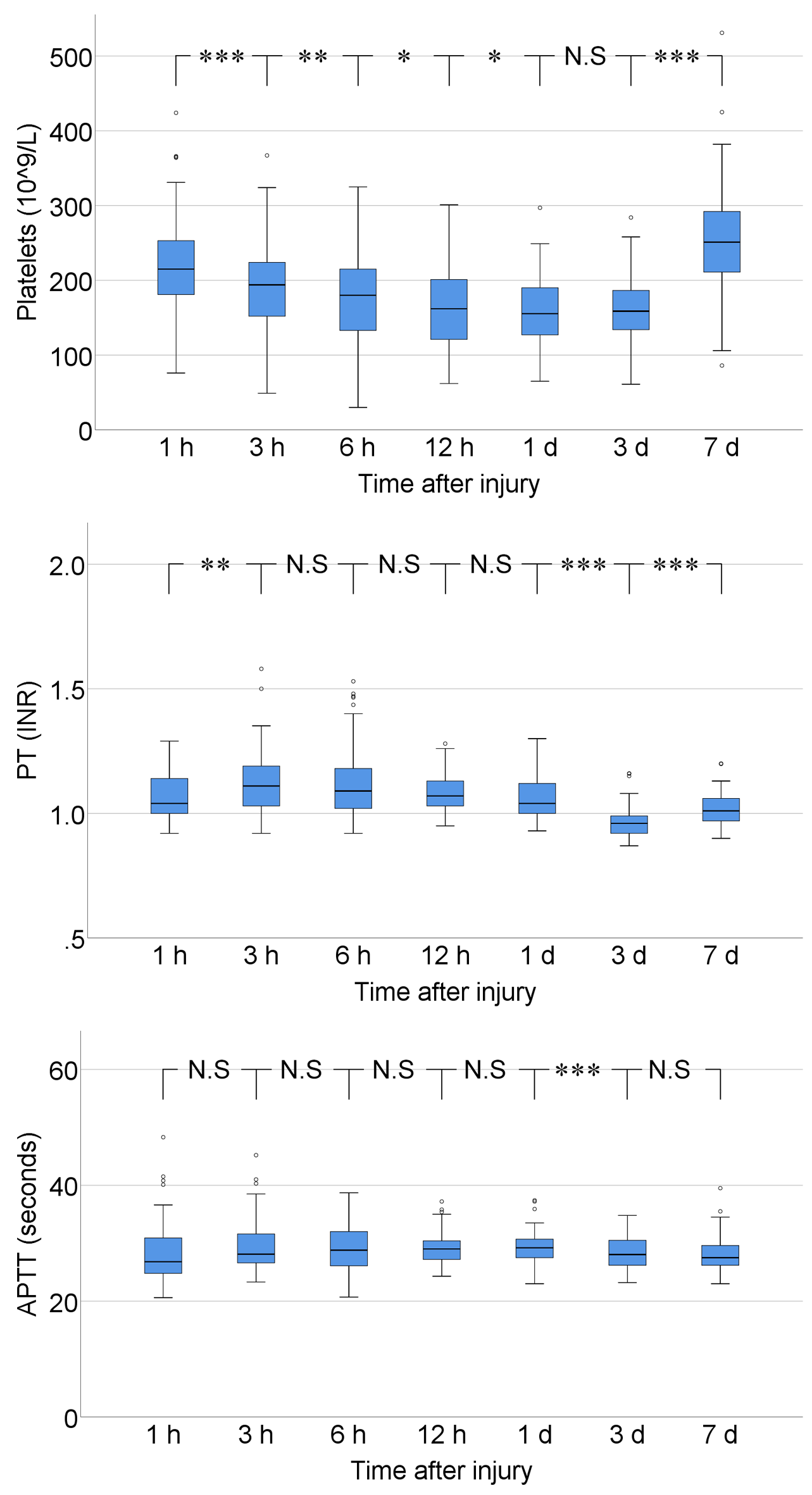


**Suppl. Fig. 1** Boxplots showing platelet count, prothrombin time (PT), activated partial thromboplastin time (APTT) of all patients on admission and 3 h, 6 h, 12 h, 1 d, 3 d, and 7 d after traumatic brain injury. **p* < 0.05, ***p* < 0.01, ****p* < 0.001, N.S = Not Significant, INR = international normalised ratio.


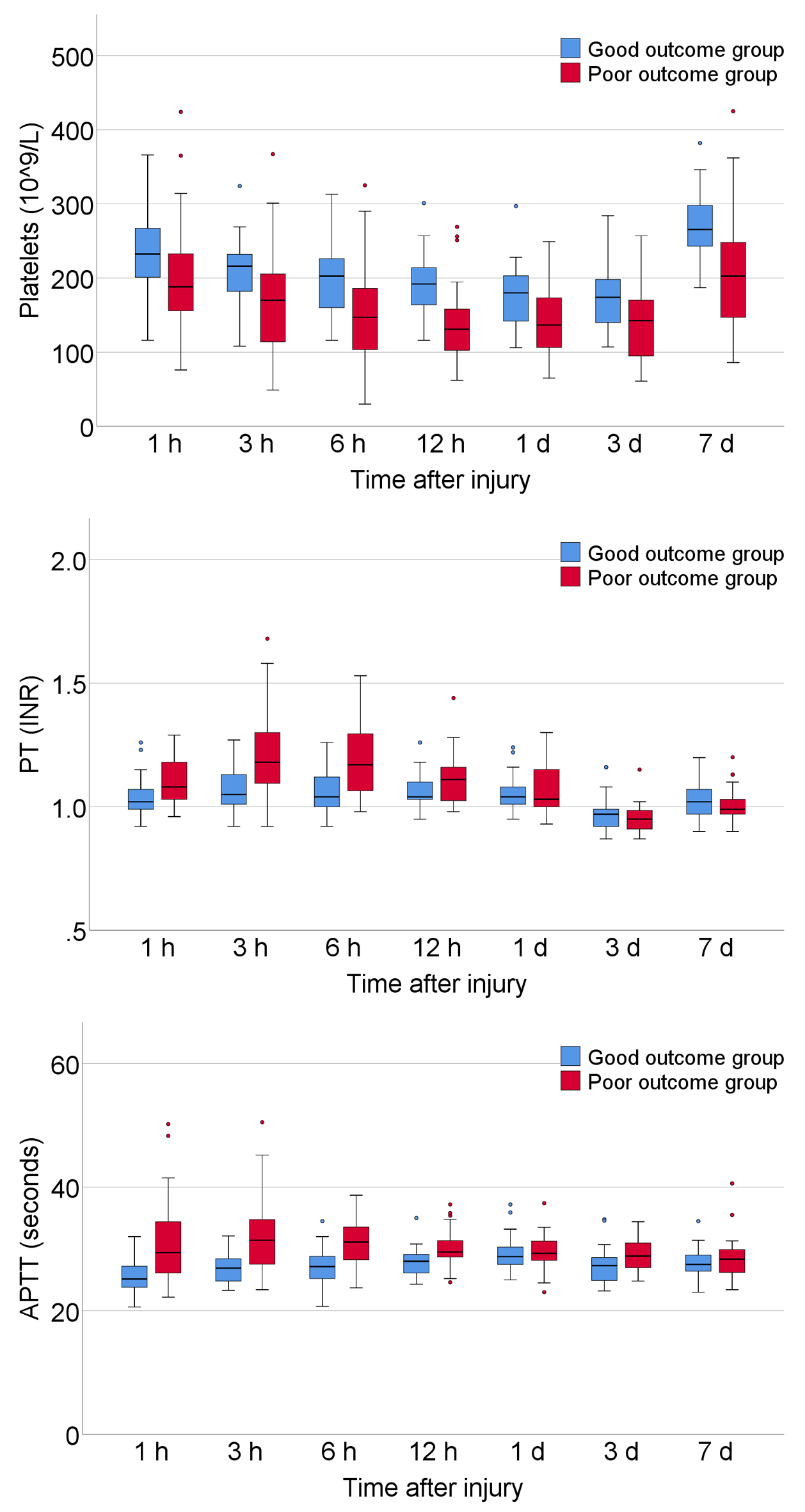


**Suppl. Fig. 2** Boxplots showing platelet count, prothrombin time (PT), activated partial thromboplastin time (APTT) of cases with good outcome and poor outcome on admission and 3 h, 6 h, 12 h, 1 d, 3 d, and 7 d after traumatic brain injury. INR = international normalised ratio.
